# The EC-HDA9 complex rhythmically regulates histone acetylation at the *TOC1* promoter in *Arabidopsis*

**DOI:** 10.1038/s42003-019-0377-7

**Published:** 2019-04-23

**Authors:** Kyounghee Lee, Paloma Mas, Pil Joon Seo

**Affiliations:** 1 Department of Biological Sciences, Sungkyunkwan University, Suwon, 16419 Republic of Korea; 2 Center for Research in Agricultural Genomics (CRAG), Consortium CSIC-IRTA-UAB-UB, Parc de Recerca Universitat Autònoma de Barcelona (UAB), Bellaterra (Cerdanyola del Vallés), Barcelona, Spain; 3 Consejo Superior de Investigaciones Científicas (CSIC), Barcelona, Spain; 4 Department of Chemistry, Seoul National University, Seoul, 08826 Republic of Korea; 5 Plant Genomics and Breeding Institute, Seoul National University, Seoul, 08826 Republic of Korea

**Keywords:** Molecular biology, Plant sciences

## Abstract

Circadian clocks are conserved time-keeper mechanisms in some prokaryotes and higher eukaryotes. Chromatin modification is emerging as key regulatory mechanism for refining core clock gene expression. Rhythmic changes in histone marks are closely associated to the *TIMING OF CAB EXPRESSION 1* (*TOC1*) *Arabidopsis* clock gene. However, the chromatin-related modifiers responsible for these marks remain largely unknown. Here, we uncover that the chromatin modifier HISTONE DEACETYLASE 9 (HDA9) and the Evening complex (EC) component EARLY FLOWERING 3 (ELF3) directly interact to regulate the declining phase of *TOC1* after its peak expression. We found that HDA9 specifically binds to the *TOC1* promoter through the interaction with ELF3. The EC-HDA9 complex promotes H3 deacetylation at the *TOC1* locus, contributing to suppressing *TOC1* expression during the night, the time of EC function. Therefore, we have identified the mechanism by which the circadian clock intertwines with chromatin-related components to shape the circadian waveforms of gene expression in *Arabidopsis*.

## Abstract (web summary)

Kyounghee Lee et al. show that a chromatin modifier HDA9 and a component of the Evening Complex, ELF3, cooperatively regulate the declining phase of Arabidopsis clock gene *TOC1*. This study illustrates how the circadian clock cooperates with histone deacetylase to regulate the circadian gene expression in Arabidopsis.

## Introduction

Circadian clocks generate biological rhythms with a period of 24 h and control plant growth and development in synchronization with the environmental cycles. Multiple transcriptional feedback loops define the basic architecture of the plant circadian clock. In *Arabidopsis*, two single-MYB transcription factors, CIRCADIAN CLOCK–ASSOCIATED 1 (CCA1) and LATE ELONGATED HYPOCOTYL (LHY), repress transcription of the *TOC1/PSEUDO RESPONSE REGULATOR 1* (*PRR1*) that in turn represses *CCA1* and *LHY* expression, constituting the central loop^1,2^. The central loop is interlocked with additional transcriptional loops. In the morning, a transcriptional repressing wave includes additional members of the PRR family including PRR5, PRR7, and PRR9 and CCA1 and LHY^3,4^. Evening-expressed clock components such as TOC1 and the EC contribute to the repression of the morning-expressed genes^5–8^. Additional transcriptional regulation also underlies circadian frameworks^9–11^. Moreover, accumulating evidence suggests that multiple layers of regulation such as alternative splicing, controlled protein turnover, and posttranslational modification further contribute to a precise rhythmic oscillation^12–14^.

Chromatin conformation influences the accessibility of transcriptional regulator(s) to the associated DNA regions. Chemical modifications at histone N-terminal tails contribute to proper packing of chromatin^15^. In particular, histone acetylation is implicated primarily in transcriptional activation^16^. Histone acetyltransferases (HATs) catalyze the addition of acetyl groups to lysine residues at histones, neutralizing positive charges and thus decreasing the affinity of histones to DNA^17^. This facilitates the accessibility of transcriptional regulators and other chromatin modifiers^18^. In contrast, histone deacetylases (HDACs) antagonize the action of HATs by removing the acetyl groups from histone^19^.

The first study relating chromatin modification and the *Arabidopsis* circadian clock identified the circadian changes in Histone 3 acetylation (H3ac) at the *TOC1* promoter. The antagonistic action of CCA1 at dawn and another single MYB clock-related transcription factor REVEILLE8/LHY-CCA1-LIKE5 (RVE8/LCL5) was found to define the hypo-acetylated and hyper-acetylated states of H3 at the *TOC1* promoter during the day^10^. Indeed, CCA1 facilitates a repressive chromatin conformation at dawn either by interfering with HAT accessibility or by recruiting HDAC activity^20^. During the day, RVE8/LCL5 antagonizes the CCA1 repressive function, favoring H3ac^10^. These counteracting functions precisely shape the waveform of *TOC1* expression^10^. The rhythmic accumulation of different histone marks was also observed at the promoters of other core clock components, including *CCA1*, *LHY*, *LUX ARRHYTHMO* (*LUX*), and *PRR*s, and correlates with their transcript accumulation^21^.

Despite the clear rhythms in histone acetylation at the *TOC1* locus, the chromatin remodeling factor(s) contributing to the rhythms of histone acetylation and its inner working mechanism remain elusive. Here, we report that HDA9, a member of the reduced potassium dependency 3 (RPD3)/HDA1 family class I HDAC^22,23^, is involved in the circadian regulation of *TOC1* by suppressing its expression. HDA9 specifically interacts with an EC component, ELF3, and the physical interaction enables HDA9 to bind to the *TOC1* promoter. HDA9 might facilitate a closed chromatin structure at the *TOC1* promoter, contributing to its declining phase of expression during the night period. These results provide an insight into how temporal regulation of histone acetylation is achieved in order to stably maintain circadian activity.

## Results

### Altered circadian oscillation in *hda9* mutant plants

Previous studies have shown that histone deacetylation is important for the rhythmic oscillation in histone acetylation at the core clock promoters^10,21,24^. As pharmacological inhibition of the RPD3/HDA1 family class I HDAC activities^25^ with TSA (Trichostatin A) affects the circadian oscillation^20,25^, we investigated their possible function within the circadian clock. Among the four members of this family (HDA6, HDA7, HDA9, and HDA19)^26^, we focused on two members not previously studied, HDA7 and HDA9.

**Fig. 1:**
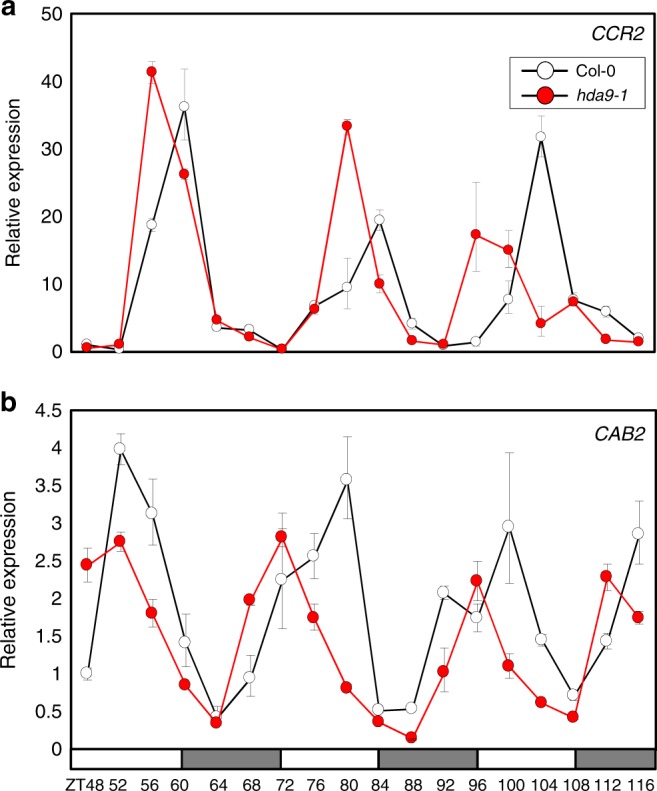
Mutation of *HDA9* alters circadian oscillation. Two-week-old seedlings grown under neutral day conditions (ND) were transferred to continuous light conditions (LL) at ZT0. Whole seedlings (*n* > 15) were harvested from ZT48 to ZT116 to analyze transcript accumulation of *CCR2* (**a**) and *CAB2* (**b**). *eIF4a* was used as the normalization control. Two technical replicates were averaged. Bars indicate the standard deviation. The white and gray boxes indicate the subjective day and night, respectively

To that end, we obtained *HDA7* and *HDA9*-deficient mutants, *hda7–2* and *hda9–1*, and analyzed rhythmic expression of the circadian oscillator gene, *CCA1*. Quantitative real-time RT-PCR (RT-qPCR) analysis revealed that the circadian expression of *CCA1* was unaffected in *hda7–2* mutant compared with wild type (Supplementary Fig. 1). However, the lack of *HDA9* led to clear alterations in *CCA1* circadian oscillation (Supplementary Figs. 1 and 2). *CCA1* phase appeared advanced, which suggests a possible shortening of the circadian period in *hda9–1* (Supplementary Fig. 1). Consistently, expression of circadian output genes, *COLD, CIRCADIAN RHYTHM, AND RNA BINDING* (*CCR2*) and *CHLOROPHYLL A/B BINDING–PROTEIN 2* (*CAB2*), and several circadian oscillator genes displayed a similar pattern with a period shortening and advanced rhythmic phase (Fig. 1a, b; also see Supplementary Figs. 3–5). Thus, HDA9 is important for proper circadian oscillation.

### Binding of HDA9 to the *TOC1* promoter

**Fig. 2:**
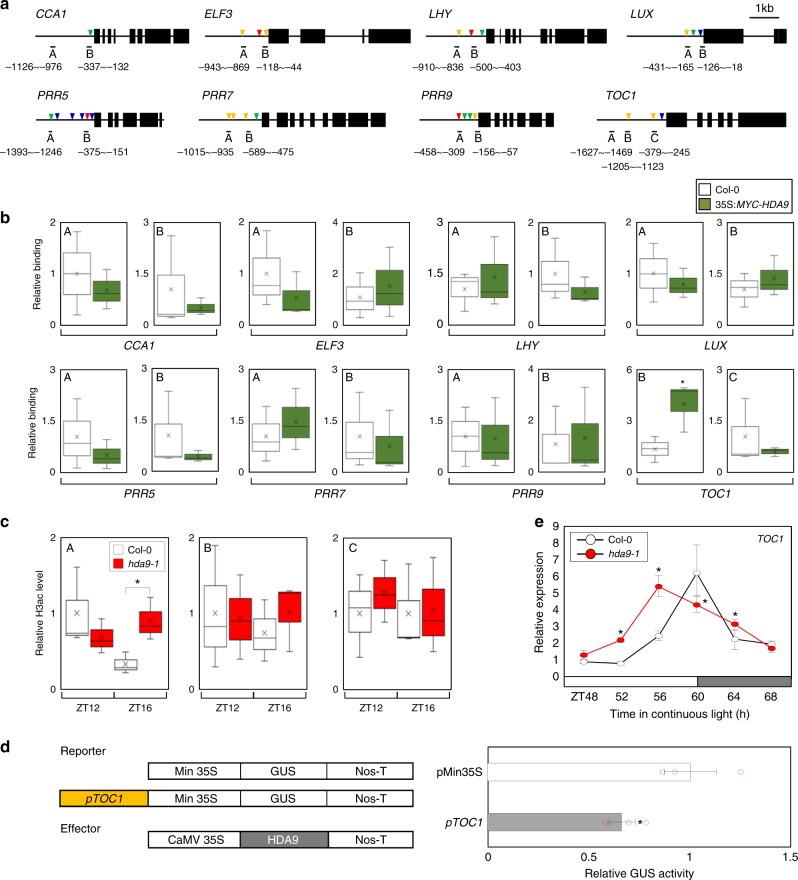
HDA9 associates with *TOC1* promoter to catalyze H3 deacetylation. **a** Genomic structures of core clock genes. Exons are represented by black boxes. Underbars indicate the regions amplified by PCR after chromatin immunoprecipitation (ChIP). Red, blue, green, and yellow arrowheads represent CBS, EE, G-box, and LBS motifs, respectively. **b** Binding of HDA9 to clock gene promoters. Two-week-old plants entrained with ND cycles were subjected to LL. Plants were harvested at ZT16 for ChIP analysis with anti-MYC antibody. Enrichment of fragmented genomic regions was analyzed by ChIP-qPCR. Biological triplicates were averaged and statistically analyzed with Student’s *t*-test (*P < 0.05; difference between Col-0 and 35 S:*MYC-HDA9* plants). Bars indicate the standard error of the mean. **c** H3ac levels at the *TOC1* locus in *hda9–1*. Two-week-old plants entrained with ND cycles were subjected to LL. Plants were harvested at ZT12 and ZT16 for ChIP analysis with anti-H3ac antibody. Biological triplicates were averaged and statistically analyzed with Student’s *t*-test (*P < 0.05; difference between Col-0 and *hda9–1* plants). Bars indicate the standard error of the mean. **d** Transient expression assays. The effector and reporter constructs were coexpressed into *Arabidopsis* protoplasts. The GUS activities were measured fluorimetrically. Biological triplicates were averaged and statistically analyzed with Student’s *t*-test (*P < 0.05). Bars indicate the standard error of the mean. **e** Expression of *TOC1* in *hda9–1*. Two-week-old seedlings grown under ND were transferred to LL at ZT0. Whole seedlings were harvested from ZT48 to ZT68. *eIF4a* was used as the normalization control. Two technical replicates were averaged and statistically analyzed with Student’s *t*-test (*P < 0.05; difference between Col-0 and *hda9–1* plants). Bars indicate the standard deviation. The white and gray boxes indicate the subjective day and night

We next aimed to identify whether HDA9 regulation of circadian gene expression occurs through direct binding to a core clock locus. To that end, we performed chromatin immunoprecipitation (ChIP) assays using 35 S:*MYC-HDA9* transgenic plants. ChIP enrichment was examined by quantitative PCR (qPCR) in the promoter regions of selected clock genes, which include key clock-related *cis*-elements, such as CCA1-binding site (CBS), evening element (EE), G-box, and/or LUX binding site (LBS) (Fig. 2a)^5,7,27,28^, in which H3ac levels are known to be rhythmically oscillating^21^. Our results showed no significant ChIP amplification except at the *TOC1* promoter (Fig. 2b). HDA9 specifically associated with a promoter region at the *TOC1* locus (Fig. 2b), but not within *TOC1* coding region (Supplementary Fig. 6).

To further explore the circadian function of HDA9, we examined its possible oscillatory expression, but found that the transcript accumulation of *HDA9* did not significantly oscillate throughout a circadian cycle (Supplementary Fig. 7). Thus, *HDA9* mRNA expression is not circadianly regulated. We nevertheless asked whether HDA9 might rhythmically bind to the *TOC1* promoter. Notably, binding of HDA9 was observed at Zeitgeber Time 16 (ZT16) (Fig. 2b and Supplementary Fig. 8), when *TOC1* expression is rather low^1^. No obvious amplification was observed at other time points examined (Supplementary Fig. 8).

HDA9 protein biochemically catalyzes the removal of acetyl groups from histone H3 proteins^22,23^. Since HDA9 binds to the *TOC1* promoter (Fig. 2b), we hypothesized that HDA9 might contribute to changes in H3ac accumulation at the *TOC1* locus. ChIP assays with an anti-H3ac antibody revealed that H3ac accumulation at a distal region of the *TOC1* promoter declined from ZT12 to ZT16 in wild type (Fig. 2c), consistent with the declined expression of *TOC1*^1^. However, the reduction of H3ac at the *TOC1* promoter was impaired in *hda9–1* mostly at ZT16 (Fig. 2c), when HDA9 binds to the *TOC1* promoter (Fig. 2b and Supplementary Fig. 8). HDA9 might bind to the specific promoter regions and then catalyze H3 deacetylation of neighboring sites. The spatial separation was consistent with the molecular function of other chromatin modifiers^29^.

Given that H3ac is associated with gene activation^16^, HDA9 function may facilitate *TOC1* repression. To test this hypothesis, we performed transient expression assays using *Arabidopsis* protoplasts. The *TOC1* promoter was fused to the 35S minimal promoter. The recombinant reporter plasmid and the effector construct expressing *HDA9* gene were co-transformed into *Arabidopsis* protoplasts. Co-expression of these constructs repressed the GUS activity by 40% (Fig. 2d). In addition, we also examined *TOC1* expression in *HDA9*-misexpressed plants. In *hda9–1* mutant plants, *TOC1* expression was clearly advanced, resulting in higher expression during the day and just after subjective dusk (Fig. 2e and Supplementary Fig. 9). Taken together, we propose that HDA9 is recruited to the *TOC1* promoter after its peak time and represses *TOC1* expression by promoting H3 deacetylation.

### Protein–protein interaction of HDA9 and ELF3

**Fig. 3:**
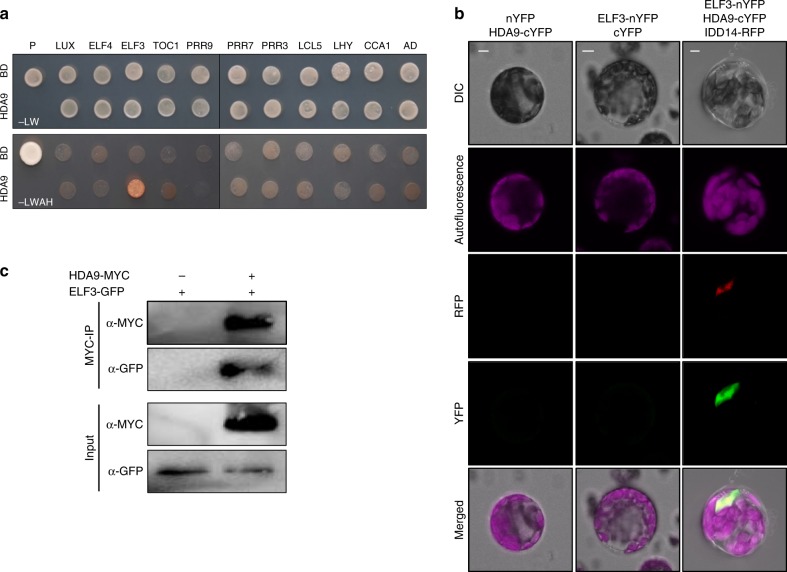
HDA9 interacts with EC. **a** Yeast-two-hybrid assays. Yeast-two-hybrid assays were performed with the HDA9 proteins fused to the DNA-binding domain (BD) of GAL4 and clock components fused with the transcriptional activation domain (AD) of GAL4 for analysis of interactions. Interactions were examined by cell growth on selective media. -LWHA indicates Leu, Trp, His, and Ade drop-out plates. -LW indicates Leu and Trp drop-out plates. GAL4 was used as a positive control. **b** BiFC assays. Partial fragments of YFP protein were fused with HDA9 and ELF3 and co-expressed in *Arabidopsis* protoplasts. IDD14-RFP was used as a nucleus marker. Scale bar, 10 µm. **c** Coimmunoprecipitation assays. *Agrobacterium tumefaciens* cells containing 35S:*HDA9-MYC* and 35S:*ELF3-GFP* constructs were coinfiltrated to 3-week-old *N. benthamiana* leaves. Epitope-tagged proteins were detected immunologically using corresponding antibodies. Full blot images were shown in Supplementary Fig. 10

Our results show that the HDA9 protein is recruited to the *TOC1* promoter. Given that *HDA9* is not circadianly regulated, we hypothesized that additional molecular component(s) might contribute to the rhythmic function of HDA9. As HDA9 itself does not have binding selectivity on specific DNA regions, DNA-binding proteins may be required for specific HDA9 binding to the *TOC1* promoter. To examine this hypothesis, we carried out yeast-two-hybrid assays with expression constructs containing core clock transcription factors. The HDA9-GAL4 DNA binding domain fusion construct was co-expressed with a construct expressing a clock gene fused in-frame to the 3’-end of GAL4 activation domain in yeast cells. Cell growth on the selective medium revealed that HDA9 specifically interacted with ELF3 (Fig. 3a), but not with other examined clock proteins (Fig. 3a).

To support the interaction of HDA9 with ELF3 in vivo, we performed bimolecular fluorescent complementation (BiFC) analysis using *Arabidopsis* protoplasts. The *HDA9* cDNA sequence was fused in-frame to the 5’-end of a gene sequence encoding the C-terminal half of YFP (cYFP), and the *ELF3* gene was fused in-frame to the 5’-end of a sequence encoding the N-terminal half of YFP (nYFP). The fusion constructs were then transiently co-expressed in *Arabidopsis* protoplasts. The HDA9-ELF3 combination was able to visualize yellow fluorescence, which was exclusively detected in the nucleus (Fig. 3b). Moreover, in planta interactions of HDA9 and ELF3 were also verified (Fig. 3c and Supplementary Fig. 10). These results indicate that HDA9 associates with ELF3 to repress *TOC1* expression.

### The EC facilitates HDA9 binding to the *TOC1* promoter

**Fig. 4:**
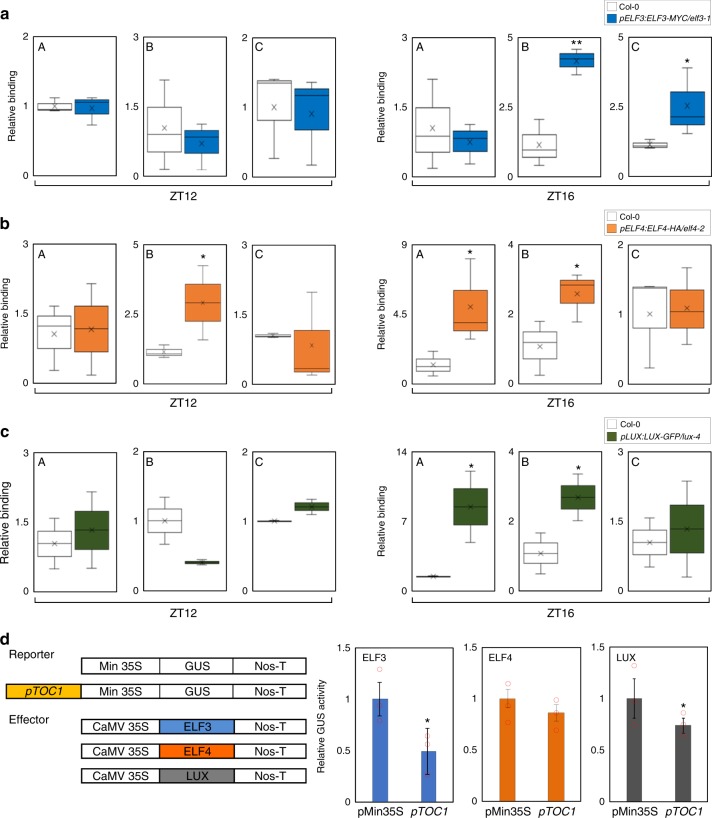
EC directly binds to the *TOC1* promoter. **a**–**c** Binding of ELF3, ELF4, and LUX to the *TOC1* locus. Two-week-old plants entrained with ND cycles were subjected to LL. Plants were harvested at ZT12 and ZT16 for ChIP analysis. Enrichment of fragmented genomic regions was analyzed by ChIP-qPCR (see Fig. 2a). Two biological replicates were averaged and statistically analyzed with Student’s *t*-test (*P < 0.05, **P < 0.01). Bars indicate the standard error of the mean. **d** Effects of ELF3, ELF4, and LUX on *TOC1* repression. The effector and reporter constructs were transiently coexpressed in *Arabidopsis* protoplasts. The GUS activities were measured fluorimetrically. Biological triplicates were averaged and statistically analyzed with Student’s *t*-test (*P < 0.05). Bars indicate the standard error of the mean

Since ELF3 is a key component of the EC^30^, HDA9 could be associated with the EC. This is in agreement with the temporal association of HDA9 to *TOC1*, because EC function is highly relevant at ZT16^30^. However, it is currently unknown whether the EC indeed associates with the *TOC1* promoter. To test this possibility, we employed *pELF3:ELF3-MYC/elf3–1*, *pELF4:ELF4-HA/elf4–2*, and *pLUX:LUX-GFP/lux-4* transgenic plants^30^. ChIP assays were performed with epitope-tagged transgenic plants immunoprecipitated with corresponding antibodies. DNA bound to epitope-tagged proteins was analyzed by qPCR assays. The ChIP-qPCR analysis showed that the *TOC1* promoter was enriched by ELF3, ELF4, and LUX (Figs. 4a–c), also in the regions where HDA9 is recruited (Fig. 2a, b). Furthermore, binding of EC components to the *TOC1* promoter occurred mainly at ZT16 (Fig. 4a–c), which is consistent with the timing of HDA9 binding (Fig. 2b).

Direct binding of EC to the *TOC1* promoter most likely allows transcriptional repression of *TOC1*. To verify the possibility, we conducted transient expression assays with the *TOC1* promoter fused to the 35S minimal promoter. The recombinant reporter plasmid and the effector construct expressing *ELF3*, *ELF4*, or *LUX* were co-transformed into *Arabidopsis* protoplasts. As expected, they bound to the *TOC1* promoter and repressed its expression (Fig. 4d), although they had different transcriptional repressive activity. Given that EC components are known to have overlapped and separate biological functions, ELF3 and LUX may play a particular role in *TOC1* regulation.

**Fig. 5:**
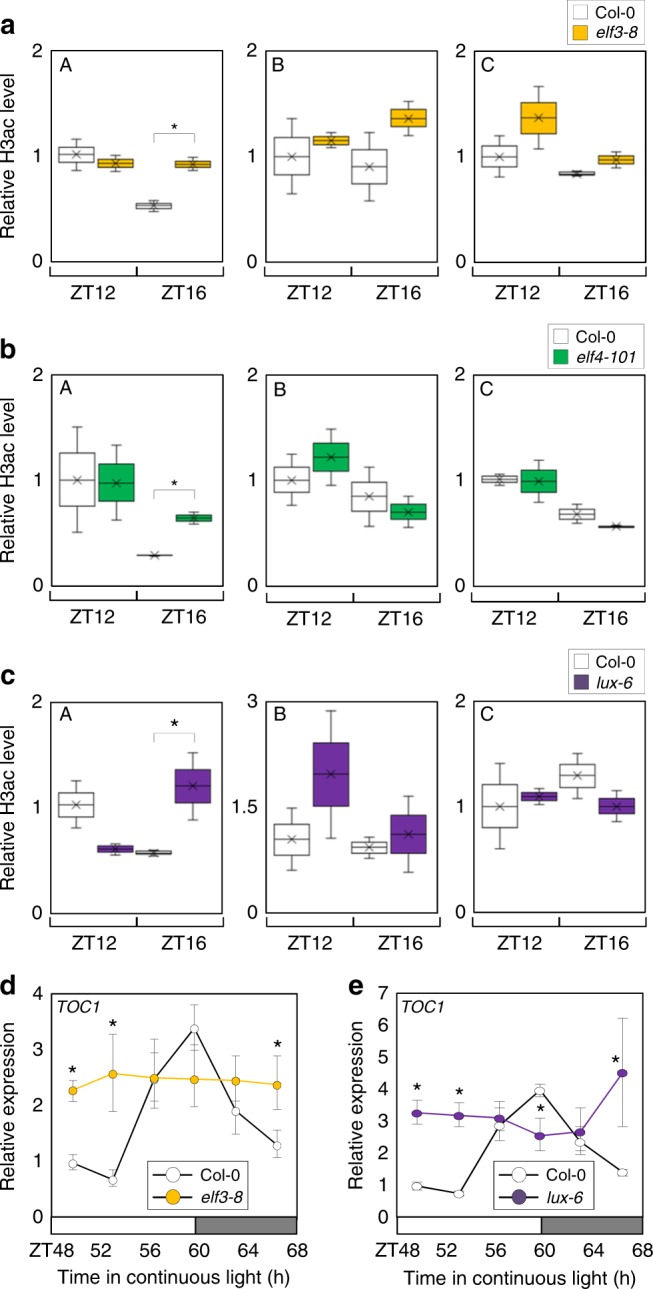
EC represses *TOC1* expression by promoting H3 deacetylation. **a**–**c** Elevated H3ac levels at *TOC1* locus in EC mutants. Two-week-old plants entrained with ND cycles were subjected to LL. Plants were harvested at ZT12 and ZT16 for ChIP analysis with anti-H3ac antibody. Two biological replicates were averaged and statistically analyzed with Student’s *t*-test (*P < 0.05). Bars indicate the standard error of the mean. **d** and **e** Expression of *TOC1* in *elf3–8* and *lux-6*. Two-week-old seedlings grown under ND were transferred to LL at ZT0. Whole seedlings were harvested from ZT48 to ZT68. *eIF4a* was used as the normalization control. Two technical replicates were averaged and statistically analyzed with Student’s *t*-test (*P < 0.05). Bars indicate the standard deviation. The white and gray boxes indicate the subjective day and night

Since the EC recruits HDA9 to repress gene expression through H3 deacetylation, we measured H3ac levels at the *TOC1* promoter in *elf3–8*, *elf4–101*, and *lux-6* mutants. ChIP-qPCR analysis revealed that H3 deacetylation at a distal region of the *TOC1* promoter was impaired at ZT16 in all mutant examined (Fig. 5a–c), in a similar fashion to that observed for *hda9–1* mutant (Fig. 2c). In addition, we also analyzed expression of *TOC1* in *elf3–8* and *lux-6* and found that *TOC1* expression was arrhythmic and elevated at some time points, including the subjective night (ZT64-ZT68) (Fig. 5d, e; also see Supplementary Figs. 11 and 12). Consistently, *CCA1* gene expression was also arrhythmic and suppressed during the subjective day (Supplementary Fig. 13), which correlates with the elevated expression of its repressor TOC1^2^. These results suggest that the EC and HDA9 act together in the control of histone deacetylation at the *TOC1* promoter.

**Fig. 6:**
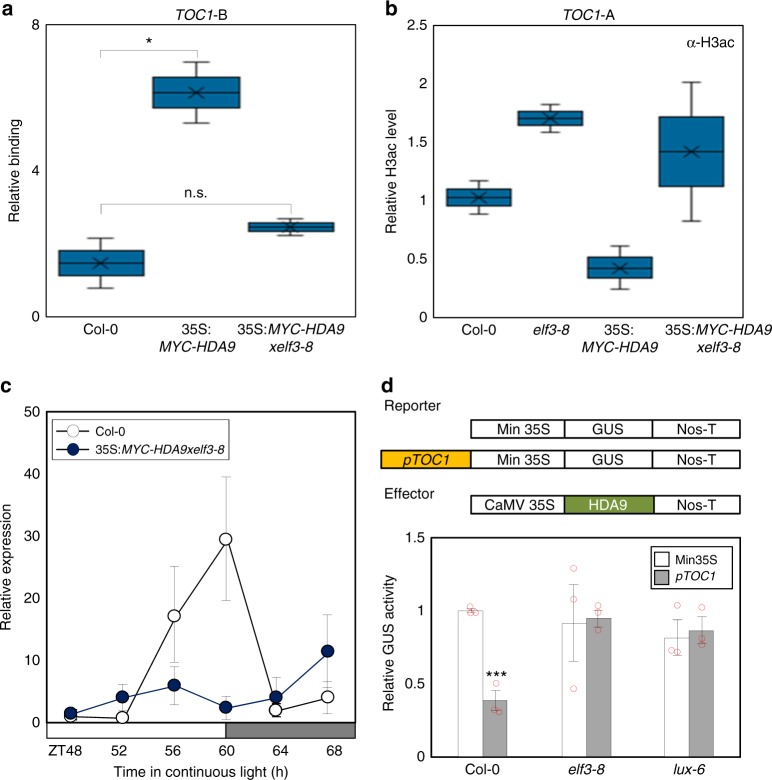
EC is required for HDA9 binding to the *TOC1* promoter. **a** Binding of HDA9 to the *TOC1* locus. **b** H3ac levels in 35S:*MYC-HDA9xelf3–8*. In **a** and **b**, two-week-old plants entrained with ND cycles were subjected to LL. Plants were harvested at ZT16 for ChIP analysis. Two biological replicates were averaged and statistically analyzed with Student’s *t*-test (*P < 0.05). Bars indicate the standard error of the mean. n.s., not significant. **c** Expression of *TOC1* in 35S:*MYC-HDA9xelf3–8*. Two-week-old seedlings grown under ND were transferred to LL at ZT0. Whole seedlings were harvested from ZT48 to ZT68. *eIF4a* was used as the normalization control. Biological triplicates were averaged. Bars indicate the standard error of the mean. The white and gray boxes indicate the subjective day and night. **d** Transient expression assays. The GUS activities were measured fluorimetrically. Biological triplicates were averaged and statistically analyzed with Student’s *t*-test (***P < 0.001). Bars indicate the standard error of the mean

The importance of EC function for HDA9 binding to the *TOC1* promoter was tested using 35S:*MYC-HDA9* transgenic plants crossed with the *elf3–8* mutant. ChIP assays using an anti-MYC antibody showed that HDA9 binding to the *TOC1* locus was reduced in the *elf3–8* mutant background compared with the wild-type background (Fig. 6a), although HDA9 protein similarly accumulated in both backgrounds (Supplementary Fig. 14). Since HDA9 promotes H3 deacetylation at the cognate regions, we also measured H3ac levels in the same plants. H3ac levels were reduced in 35S:*MYC-HDA9* plants compared with wild type, but H3 deacetylation was compromised in 35S:*MYC-HDA9xelf3–8* plants, which was equivalent to *elf3–8* (Fig. 6b). Further, 35S:*MYC-HDA9* transgenic plants displayed robust circadian expression of *TOC1*, while the amplitude was reduced compared with wild type (Supplementary Fig. 15). However, arrhythmic circadian expression was observed in 35S:*MYC-HDA9xelf3–8* (Fig. 6c), similar to EC mutants (Fig. 5d, e). Indeed, HDA9 function is most likely dependent on EC. Transient expression assays using *Arabidopsis* protoplasts revealed that HDA9 binds to the *TOC1* promoter to inhibit expression (Fig. 2d). However, the HDA9 function disappeared in *elf3–8* and *lux-6* mutants (Fig. 6d). These results suggest that HDA9 requires the ELF3, possibly EC, for binding to the *TOC1* promoter.

**Fig. 7:**
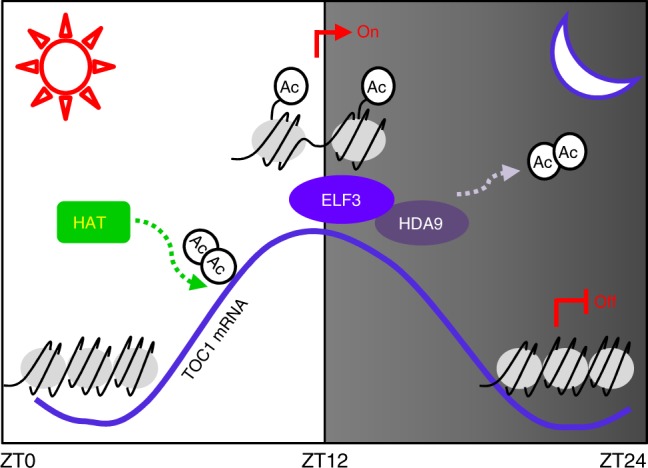
Working diagram of the EC-HDA9 complex in circadian control. Transcript accumulation of *TOC1* correlates with H3ac levels at the gene promoter. As-yet-unidentified HAT catalyzes H3ac at the *TOC1* promoter during the daytime. After peak expression, the ELF3 protein, presumably evening complex (EC), recruits HDA9. The EC-HDA9 complex binds to the *TOC1* promoter and removes H3ac at neighboring sites to decline its expression during nighttime

In summary, *TOC1* expression is regulated by rhythmic changes in histone acetylation states. A-yet-unidentified HAT activity is required for the rising phase of *TOC1* expression. After its peak expression, HDA9 is recruited to the *TOC1* promoter through the interaction with ELF3, possibly the EC. The EC-HDA9 complex facilitates H3 deacetylation at the *TOC1* locus to reach its basal expression during night period (Fig. 7). Diurnal oscillation of H3ac levels may be pervasive in core clock genes, ensuring circadian homeostasis.

## Discussion

Rhythmic expression of core clock genes is intimately associated with the levels of H3ac, especially H3K56ac and H3K9/14ac, and H3K4me3 at the gene promoters in *Arabidopsis*^21,31^. Despite the connection between chromatin modification and the circadian control, just a few responsible chromatin modifiers have been demonstrated to participate in circadian chromatin remodeling.

The SET DOMAIN GROUP 2 (SDG2)/ARABIDOPSIS TRITHORAX–RELATED 3 (ATXR3) protein establishes H3K4me3 mark at core clock gene loci to promote expression. The H3K4me3 mark inhibits clock repressor binding at the core clock promoters, facilitating transcriptional repression to target genes only at specific time-of-day^21^. Accordingly, the *SDG2*/*ATXR3*-deficient mutants exhibit a decrease in H3K4me3 accumulation, advanced clock repressor binding, and reduced amplitude of core clock gene expression^21^. In addition, the JmjC domain-containing histone demethylase JMJ30/JMJD5 is also involved in circadian homeostasis. The *JMJ30/JMJD5* gene is circadian-regulated and peaks at dusk^32,33^. The central oscillators CCA1 and LHY directly bind to and repress *JMJ30/JMJD5*. Then, JMJ30/JMJD5 reciprocally regulates expression of *CCA1* and *LHY*^32^.

A couple of HDACs have been shown to be implicated in the *Arabidopsis* circadian system. PRR5, PRR7, and PRR9 directly repress *CCA1* and *LHY*^34^. Notably, PRRs interact with TOPLESS/TOPLESS RELATED PROTEINS (TPL/TPRs), as well as HDA6 and HDA19^34^. Consistently, inhibition of the TPL activity compromises transcriptional repression activities of PRR5, PRR7, and PRR9, lengthening circadian period^34^. The potent HDAC inhibitor TSA activates *CCA1* expression, but this activation is impaired in the *tpl-1* mutant, demonstrating that HDAC regulation is largely dependent on TPL^34^.

We here show that HDA9 regulates circadian oscillation by modulating *TOC1* expression. HDA9 binds to the *TOC1* promoter and stimulates H3 deacetylation to suppress *TOC1* expression. HDA9 interacts with the EC component ELF3, and rhythmic binding of HDA9 to the *TOC1* promoter is defined by the EC. Accordingly, although *HDA9* has no obvious rhythmic expression pattern, the EC-HDA9 interactions facilitate temporal regulation of *TOC1*. Consistent with the fact that the EC is expressed highly during night period^35^, the EC-HDA9 complex is relevant in the declining phase of *TOC1* expression. Nonetheless, many questions remain to be answered. Considering the variable alteration patterns of circadian gene expression in *hda9–1* mutant, EC and HDA9 are extensively interconnected with other circadian and chromatin-related components, accounting for dynamic contribution to circadian oscillation. Moreover, it is sometimes observed the anti-phase of binding regions and histone modifications^29,36^. While HDA9 binds to a proximal region of the *TOC1* promoter, changes in histone acetylation are drastically observed in a distal region. This may be due to propagation and expansion of epigenetic modification of chromatin. Chromatin remodeling components that regulate epigenetic contexts on chromatin would also be involved, and thus the responsible protein components should be further elucidated. Future studies will give a comprehensive view of circadian-chromatin networks. Altogether, these results exemplify a mechanism by which chromatin factors engage with clock components to rhythmically modulate chromatin conformation and hence transcriptional oscillation.

The HDA9 protein is implicated in plant developmental processes. *HDA9* is highly expressed in dry seeds and represses seedling traits to maintain seed dormancy^37^. The *hda9* mutants show reduced seed dormancy with higher expression of genes responsible for photosynthesis and photoautotroph, such as RuBisCO and RuBisCO activase (RCA)^37^. The HDA9 action is antagonistic to HDA6 and HDA19, which repress embryonic properties in germinating seeds^37^, balancing the proper transition from seed to autotropic seedling.

The *HDA9*-deficient mutants also exhibit early flowering phenotypes under short day conditions^38,39^. HDA9 binds to the *AGAMOUS–LIKE 19* (*AGL19*) promoter and negatively regulates its transcription through the alterations in H3K9 and H3K27 acetylation levels at the *AGL19* locus^39^. Consequently, the HDA9-AGL19 module mainly regulates *FLOWERING LOCUS T* (*FT*) expression, in parallel with photoperiod or autonomous pathways to prevent precocious flowering in short days^39^. The epigenetic regulation of *AGL19* by HDA9 is also relevant, in part, during vernalization process. Given that HDA9 is functional when *AGL19* is actively expressed, such in the adult stage or after vernalization^39^, this HDAC protein is able to associate with transcriptionally active genes to reset the chromatin state. Accumulating evidence further supports that the HDA9-POWERDRESS (PWR) complex stimulates H3 deacetylation at a genome-wide level and is implicated in additional developmental processes, such as floral stem cell fate, stress responses, and aging^22,23^.

Notably, HDA9 activity can be diurnally gated. HDACs often form a massive protein complex containing for instance Sin3, RPD3-type HDAC, SIN3-ASSOCIATED POLYPEPTIDE 18 (SAP18) and SAP30^40^. Homologs of each component are present in *Arabidopsis*: six SIN3-Like (SNL1-SNL6), four RPD3 HDACs, one SAP18, and two SAP30 function-relateds (AFR1 and AFR2)^40^. The *Arabidopsis* Sin3-HDAC complex is crucial for diurnal control of *FT*^40^. This complex accumulates at dusk and is recruited to *FT* promoter to ensure periodic histone deacetylation. Dynamic cycles of H3 acetylation and deacetylation ensure adequate levels of the gene transcription and thereby prevent precocious flowering in the premature inductive conditions^40^. This study further supports periodic functions of HDA9 in controlling circadian gene expression. H3ac states can be reversible and account for stably oscillating gene expression. Taken together, the HDA9 protein seems to integrate temporal information to regulate oscillator gene expression and a variety of physiological output processes in order to optimize plant growth and development.

## Methods

### Plant materials and growth conditions

*Arabidopsis thaliana* (Columbia-0 ecotype) was used for all experiments unless otherwise specified. Plants were grown under neutral-day conditions (NDs; 12-h light/12-h dark cycles) with cool white fluorescent light (100 μmol photons m^−2^ s^−1^) at 22 °C. The *elf3–8*, *elf4–101*, *hda7–2*, and *hda9–1* were previously reported^39,41–43^. The *lux-*6 mutant (SALK-022315) was obtained from *Arabidopsis* Biological Resource Center (ABRC). To produce transgenic plants overexpressing the *HDA9* gene, a full-length cDNA was subcloned into the binary pBA002 vector under the control of the CaMV 35 S promoter. *Agrobacterium tumefaciens*-mediated *Arabidopsis* transformation was then performed.

### Quantitative real-time RT-PCR analysis

Plants grown under conditions/ZT indicated were harvested and ground in liquid nitrogen. Total RNA was extracted by mixing the tissue powder with 700 μl of TRI reagent (TAKARA Bio, Singa, Japan). The extraction mixture was incubated at 65 °C for 5 min. After mixing with 700 μl of chloroform:isoamyl alcohol (24:1), the extraction mixture was centrifuged for 20 min at room temperature. The RNA in the liquid phase was precipitated at 4 °C in a final concentration of 2 M LiCl. RNA pellet was washed by 70% ethanol, briefly air dried, and suspended in distilled water. Reverse transcription (RT) was performed using Moloney Murine Leukemia Virus (M-MLV) reverse transcriptase (Dr. Protein, Seoul, South Korea) with oligo(dT18) to synthesize first-strand cDNA from 2 μg of total RNA. Total RNA samples were pretreated with an RNAse-free DNAse to remove DNA contamination.

Quantitative RT-PCR reactions were performed on the Step-One Plus Real-Time PCR System (Applied Biosystems). The PCR primers used are listed in Supplementary Table 1. The comparative C_T_ method was used to determine the relative gene expression, with the expression of *Eukaryotic translation initiation factor 4A1* (*eIF4A*) gene (At3g13920) as the internal control. All RT-qPCR reactions were performed with biological triplicates using total RNA samples extracted from three independent replicate samples.

### Yeast two-hybrid assays

Yeast two-hybrid assays were performed using the BD Matchmaker system (Clontech, Mountain View, CA, USA). The pGADT7 vector was used for the GAL4 activation domain fusion, and the pGBKT7 vector was used for the GAL4 DNA binding domain fusion. The yeast strain AH109 harboring the *LacZ* and *His* reporter genes was used. PCR products were subcloned into the pGBKT7 and pGADT7 vectors. The expression constructs were co-transformed into yeast AH109 cells, and transformed cells were selected by growth on SD/-Leu/-Trp medium and SD/-Leu/-Trp/-His/-Ade.

### Bimolecular fluorescence complementation (BiFC) assays

The *HDA9* gene was fused in-frame to the 5′ end of a gene sequence encoding the C-terminal half of EYFP in the pSATN-cEYFP-C1 vector (E3082). The *ELF3* cDNA sequences were fused in-frame to the 5′ end of a gene sequence encoding the N-terminal half of EYFP in the pSATN-nEYFP-C1 vector (E3081). Expression constructs were co-transformed into *Arabidopsis* protoplasts. Expression of the fusion constructs was monitored by fluorescence microscopy using a Zeiss LSM510 confocal microscope (Carl Zeiss, Jena, Germany).

### Chromatin immunoprecipitation (ChIP) assays

The epitope-tagged transgenic plant samples were cross-linked with 1% formaldehyde, ground to powder in liquid nitrogen, and then sonicated (15-s ON/15-s OFF for seven times, each with 5 min). The sonicated chromatin complexes were bound with corresponding antibodies. Anti-MYC (05–724, Millipore, Billerica, USA), anti-H3ac (06–599, Millipore, Billerica, USA), anti-HA (ab9110, Abcam, Cambridge, USA), and salmon sperm DNA/protein A agarose beads (16–157, Millipore, Billerica, USA) were used for chromatin immunoprecipitation. The immunoprecipitated chromatin complexes were purified by incubating with 50 μl slurry of Protein-A Sepharose (GE, 17–5280–01) pre-equilibrated with 1 mg/ml salmon sperm DNA and 1 mg/ml BSA for 3 h at 4 °C. The affinity beads bound chromatin complexes were washed with 850 μl nuclei lysis buffer for three times, LNDET buffer (0.25 M LiCl, 1% NP40, 1% sodium deoxycholate, 1 mM EDTA, pH8.0) for three times and TE buffer (10 mM Tris–HCl, 1 mM EDTA, pH8.0) for three times. Chromatin complexes were eluted from the beads with use of 200 μl elution buffer (1% SDS, 0.1 M NaHCO_3_) followed by the digestion with 2.5 ml Proteinase-K (20 mg/ml, Invitrogen, Carlsbad, CA) for overnight at 65 °C. DNA was purified using phenol/chloroform/isoamyl alcohol and sodium acetate (pH 5.2). The level of precipitated DNA fragments was quantified by quantitative real-time PCR using specific primer sets (Supplementary Table 2). Values were normalized to an internal control (*eIF4a*). Values for control plants were set to 1 after normalization against *eIF4a* for quantitative PCR analysis.

### Transient expression assays

For transient expression assays using *Arabidopsis* protoplasts, reporter and effector plasmids were constructed. The reporter plasmid contains a minimal 35S promoter sequence and the GUS-coding gene. The *TOC1* promoter was inserted into the reporter plasmid. The coding regions of *HDA9*, *ELF3*, *ELF4*, and *LUX* were subcloned into the effector vector for transient overexpression under the control of the CaMV 35 S promoter. An amount of 2.5 × 10^5^ protoplasts was co-transfected with 4 μg reporter construct, 4 μg effector construct, and 2 μg CaMV 35 S promoter-luciferase construct as the internal transfection control. Transfected cells were incubated at 22 °C overnight and harvested for LUC and GUS reporter assays.

### Reporting Summary

Further information on experimental design is available in the Nature Research Reporting Summary linked to this article.

## Supplementary information

Supplementary Information

Reporting Summary

## Data Availability

All relevant data are available from the corresponding author on request.
