# Supplementary material for: The EC-HDA9 complex rhythmically regulates histone acetylation at the TOC1 promoter in Arabidopsis
Source: Commun Biol. 2019 Apr 23;2:143. doi: 10.1038/s42003-019-0377-7 (PMC6478914; doi:10.1038/s42003-019-0377-7)
Supplement: Supplementary file 1 — Supplementary Information [file 42003_2019_377_MOESM1_ESM.pdf]

Supplementary Information

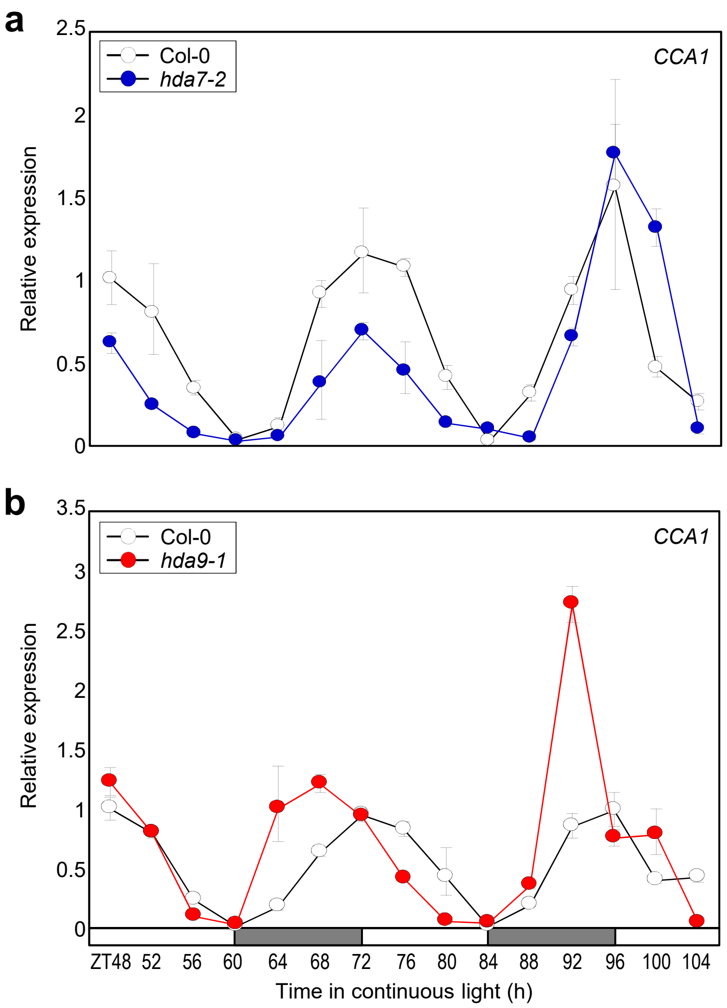

Supplementary Figure 1. Circadian expression of *CCA1* in *hda7-2* and *hda9-1*.
Two-week-old *hda7-2* **(a)** and *hda9-1* **(b)** seedlings grown under neutral day conditions (ND) were transferred to continuous light conditions (LL) at ZT0. Whole seedlings were harvested from ZT48 to 104 to analyze transcript accumulation. Technical duplicates were averaged. Bars indicate the standard deviation. The white and grey boxes indicate the subjective day and night, respectively.

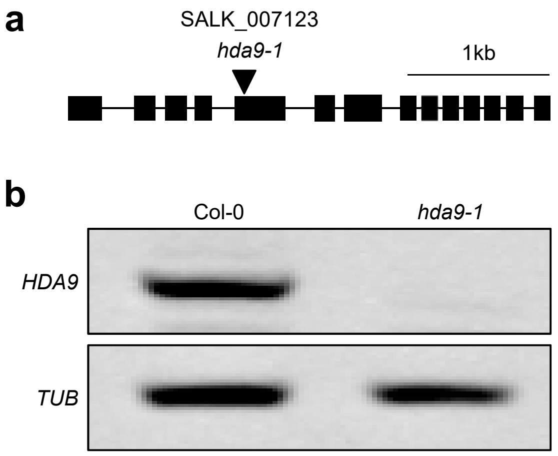

Supplementary Figure 2. Expression of *HDA9* in *hda9-1* mutant.
(a) T-DNA insertion site in *hda9-1* mutant. Black rectangles indicate exons. The arrowhead represents the T-DNA insertion site. **(b)** Transcript accumulation of *HDA9* in *hda9-1*. Transcript accumulation was analyzed by semi-quantitative RT-PCR. The *TUBULIN BETA CHAIN 2* (*TUB*) gene (At5g62690) was used as an internal control.

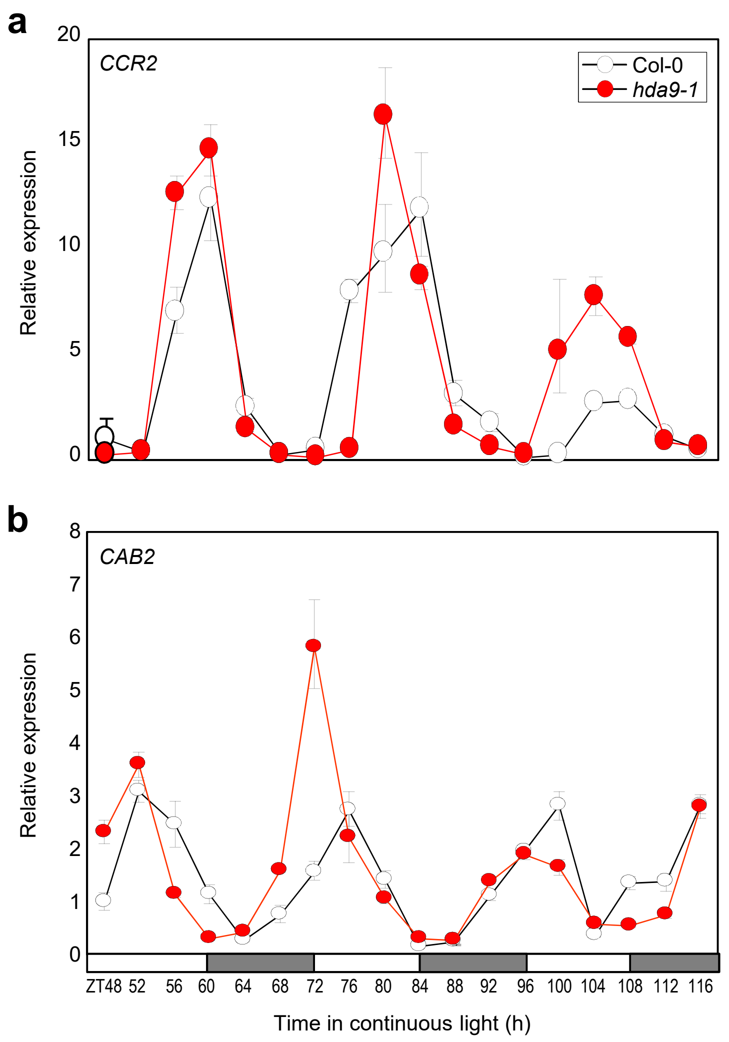

Supplementary Figure 3. Biological replicates for circadian expression of *CCR2* and *CAB2* in *hda9-1*.

Two-week-old seedlings grown under ND conditions were transferred to LL conditions at ZT0. Whole seedlings were harvested from ZT48 to ZT116 to analyze transcript accumulation of *CCR2* **(a)** and *CAB2* **(b)**. Technical duplicates were averaged. Bars indicate the standard deviation. The white and grey boxes indicate the subjective day and night, respectively. Note that these are biological replicates for Fig. 1a and 1b.

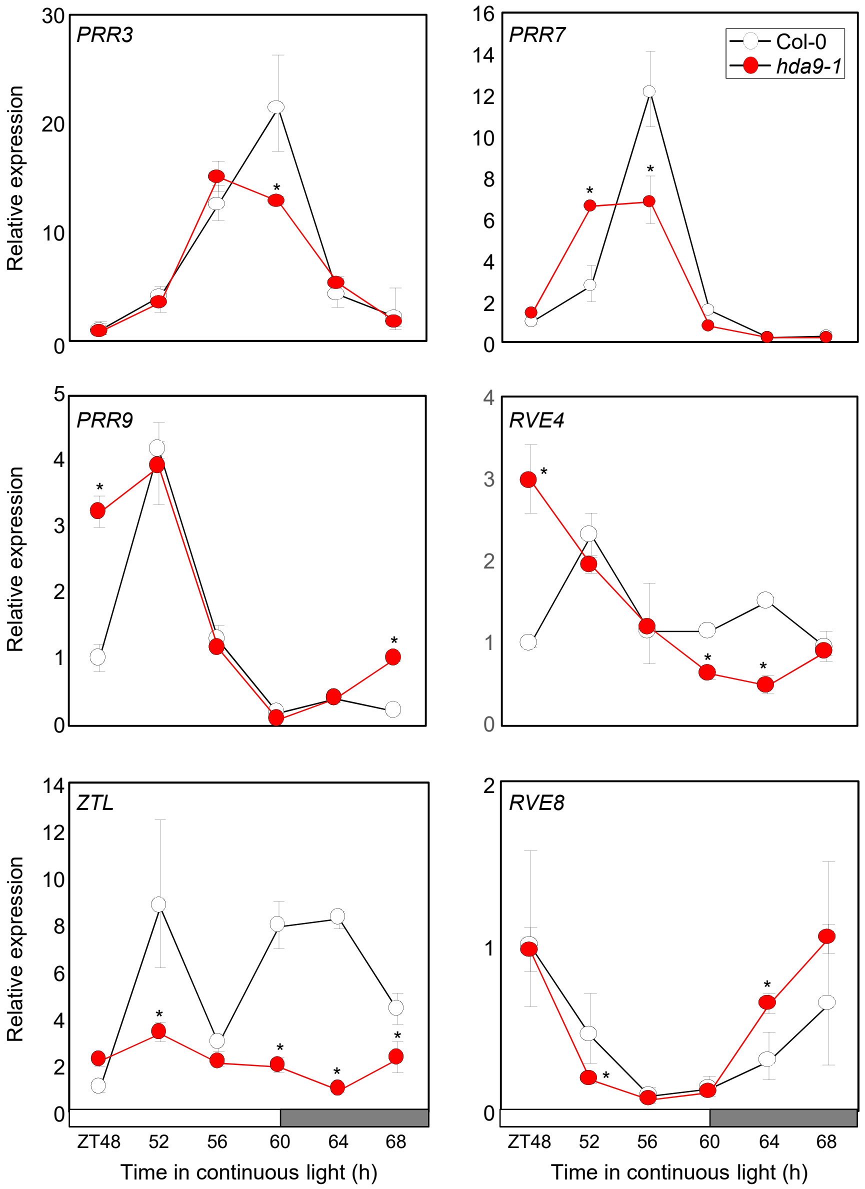

Supplementary Figure 4. Circadian expression of core clock genes in *hda9-1*.
Two-week-old seedlings grown under ND conditions were transferred to LL conditions at ZT0. Whole seedlings were harvested from ZT48 to ZT68 to analyze transcript accumulation. Technical replicates were averaged and statistically analyzed with Student's *t*-test (**P* < 0.05; difference between Col-0 and *hda9-1*). Bars indicate the standard deviation. The white and grey boxes indicate the subjective day and night, respectively.

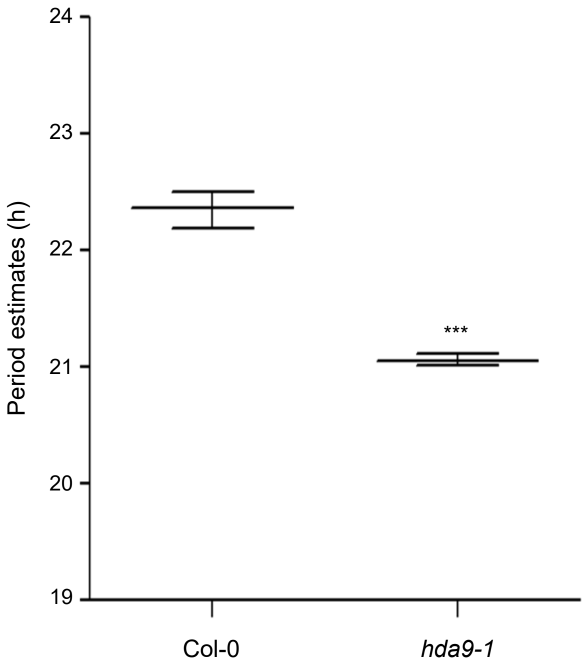

Supplementary Figure 5. Period estimates of *CCR2* expression.
Period estimates of *CCR2* expression (Fig. 1a) were calculated using fast-Fourier transformed nonlinear least squares analysis implemented in Biological Rhythms Analysis Software Suite (http://millar.bio.ed.ac.uk/PEBrown/BRASS/BrassPage.htm). Statistical significance was determined by Student's *t*-test (***$P < 0.001$). Bars indicate the standard error of the mean.

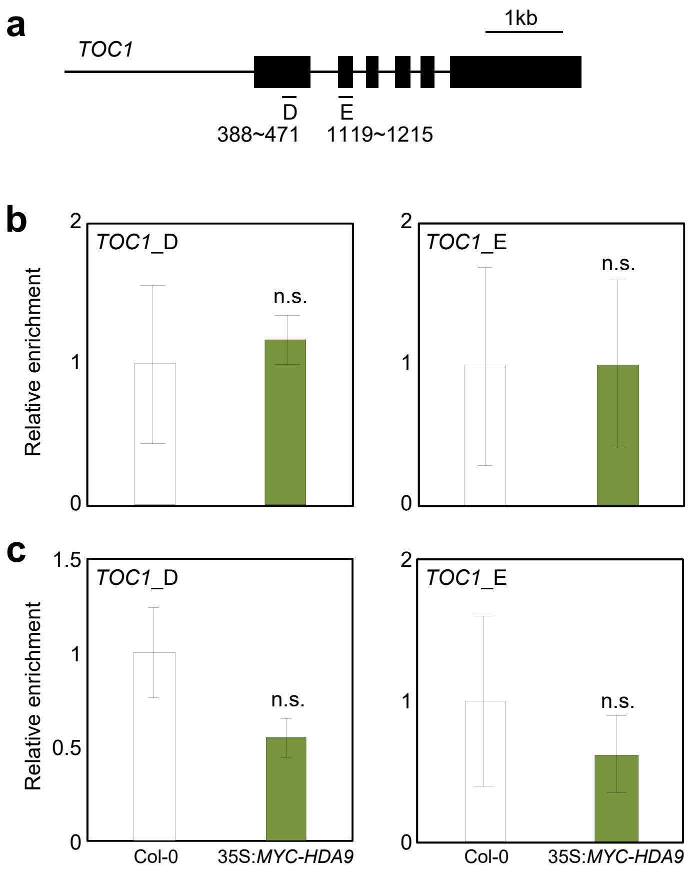

Supplementary Figure 6. Binding of HDA9 to coding regions of *TOC1*.
(a) Genomic structures of *TOC1* genes. Exons are represented by black boxes. Underbars indicate the regions amplified by PCR after chromatin immunoprecipitation (ChIP). Two-week-old plants entrained with ND cycles were subjected to LL. **(b and c)** ChIP assays. Plants were harvested at ZT12 **(b)** and ZT16 **(c)** for ChIP analysis with anti-MYC antibody. Enrichment of fragmented genomic regions was analyzed by ChIP-qPCR. Biological triplicates were averaged. Bars indicate standard error of the mean. n.s., not significant.

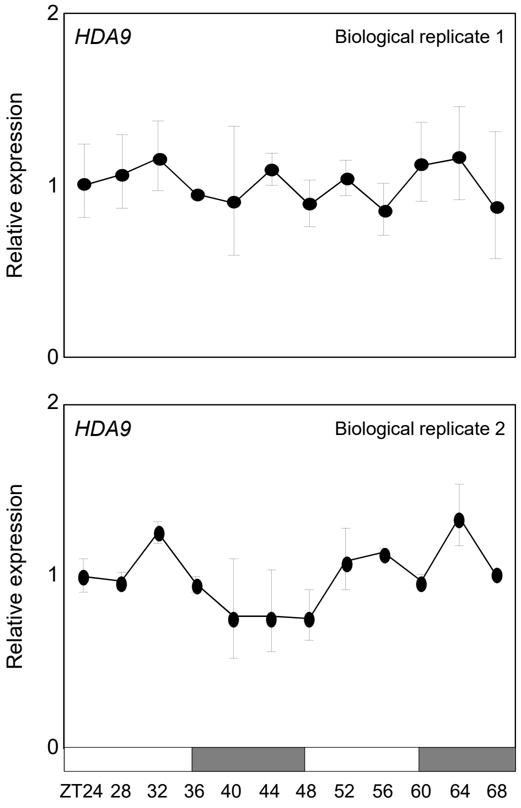

Supplementary Figure 7. Expression of *HDA9* in circadian cycles.
Twelve-day-old seedlings grown under ND were transferred to LL at ZT0. Transcript levels were determined by RT-qPCR. Biological duplicates were shown. In each biological replicate, technical duplicates were averaged. Bars indicate the standard deviation. The white and grey boxes indicate the subjective day and night, respectively.

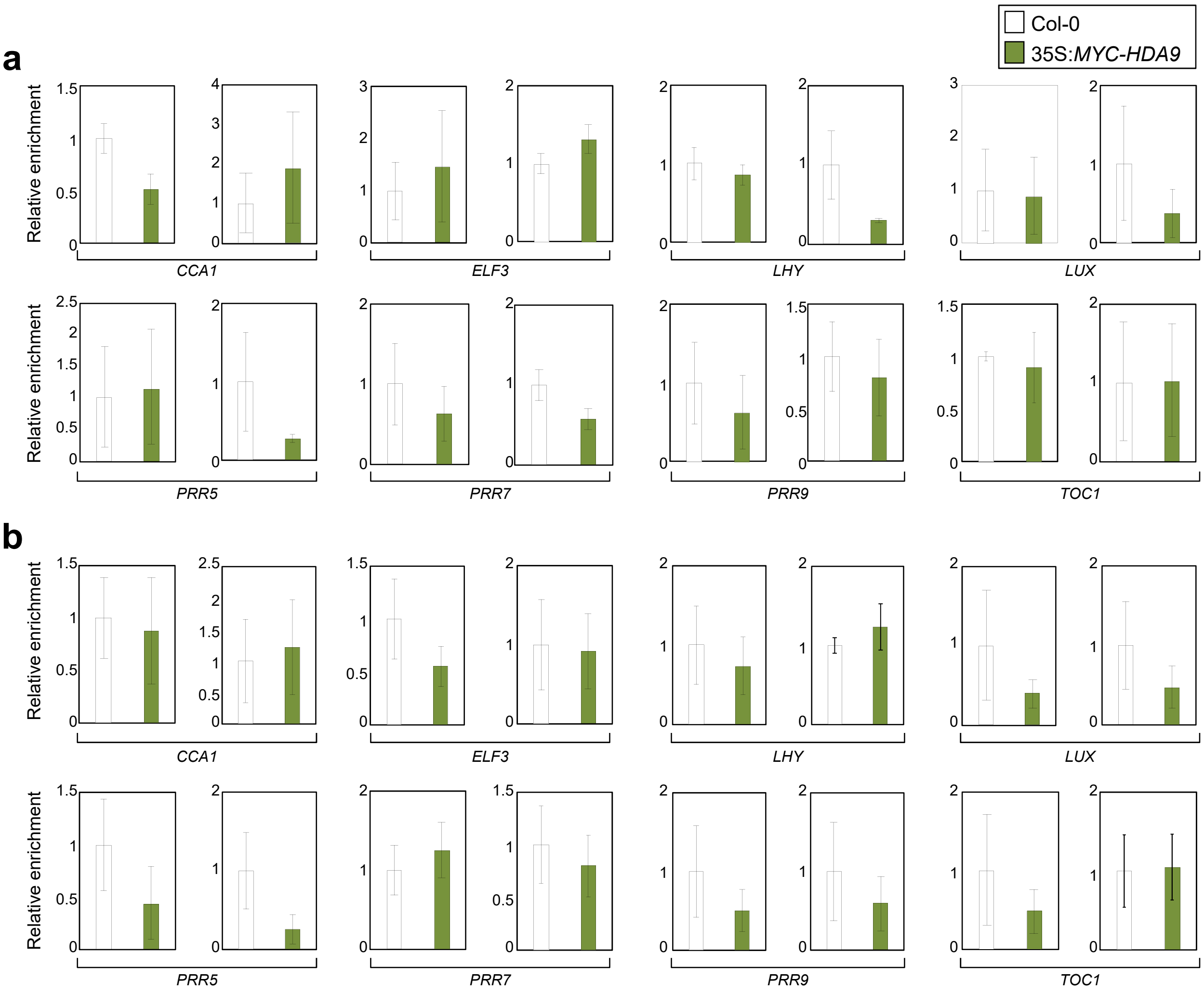

Supplementary Figure 8. Binding of HDA9 to the core clock gene promoters at ZT0 and ZT12. Two-week-old plants entrained with ND cycles were subjected to LL. Plants were harvested at ZT0 **(a)** and ZT12 **(b)** for ChIP analysis with anti-MYC antibody. Enrichment of fragmented genomic regions was analyzed by ChIP-qPCR. Biological triplicates were averaged. Bars indicate the standard error of the mean.

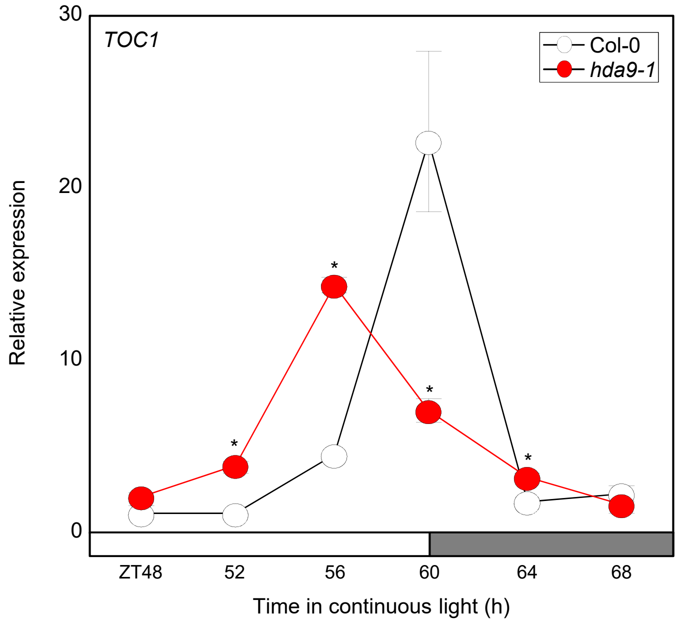

Supplementary Figure 9. Biological replicates for circadian expression of *TOC1* in *hda9-1*. Two-week-old seedlings grown under ND conditions were transferred to LL conditions at ZT0. Whole seedlings were harvested from ZT48 to ZT68 to analyze transcript accumulation. Technical duplicates were averaged and statistically analyzed with Student's *t*-test (*$P < 0.05$; difference between Col-0 and *hda9-1*). Bars indicate the standard deviation. The white and grey boxes indicate the subjective day and night, respectively. Note that this is a biological replicate for Fig. 2e.

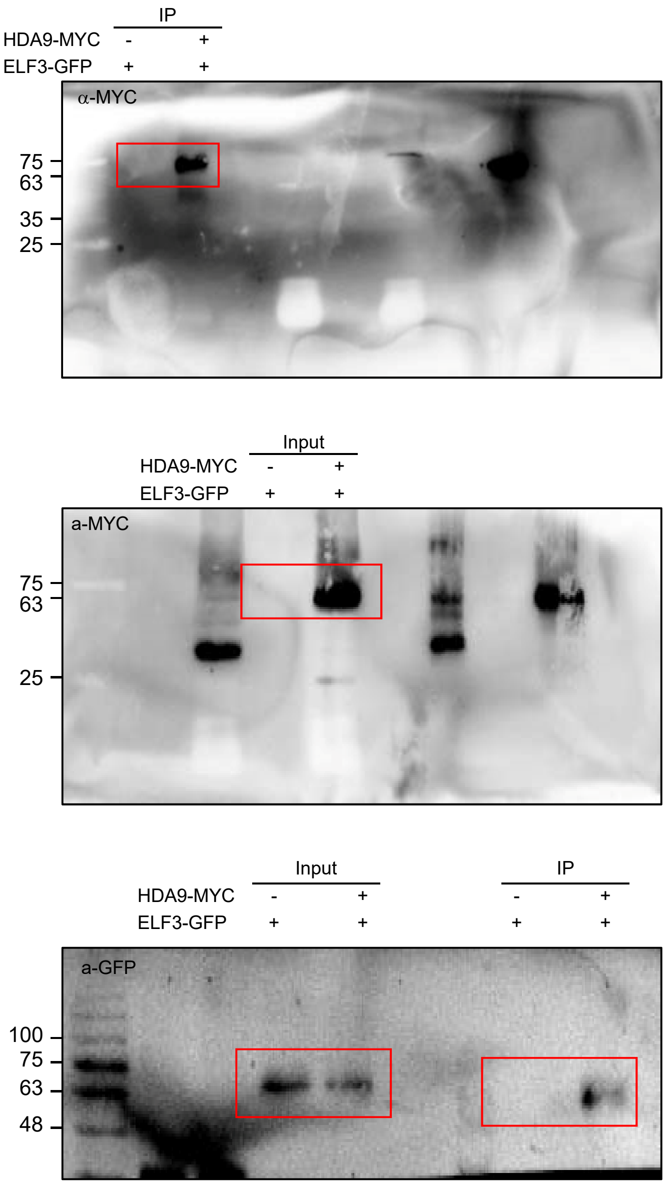

Supplementary Figure 10. Full-blot images for coimmunoprecipitation assays. *Agrobacterium tumefaciens* cells containing 35S:*HDA9-MYC* and 35S:*ELF3-GFP* constructs were coinfiltrated to 3-week-old *Nicotiana benthamiana* leaves. Epitope-tagged proteins were detected immunologically using corresponding antibodies. Note that these are full-blot images for Fig. 3c.

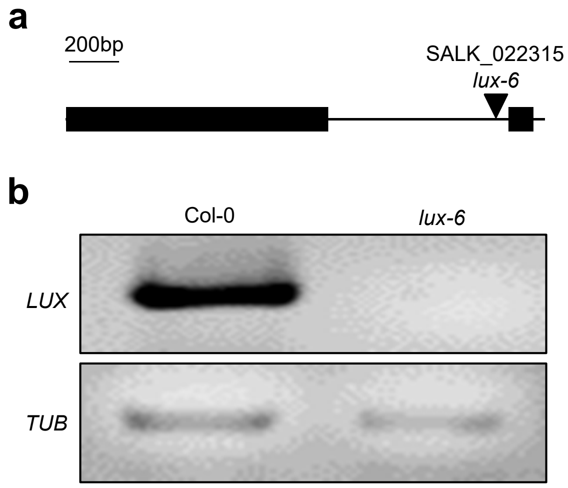

Supplementary Figure 11. Expression of *LUX* in *lux-6* mutant.
(a) T-DNA insertion site in *lux-6* mutant. Black rectangles indicate exons. The arrowhead represents the T-DNA insertion site. **(b)** Transcript accumulation of *LUX* in *lux-6*. Transcript accumulation was analyzed by semi-quantitative RT-PCR. The *TUB* gene (At5g62690) was used as an internal control.

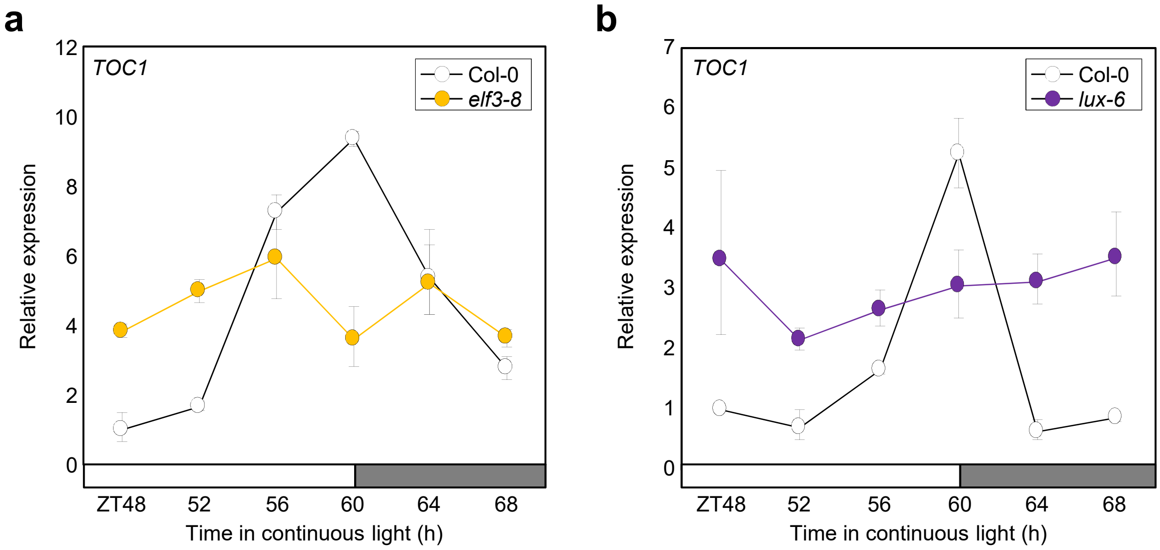

Supplementary Figure 12. Biological replicates for circadian expression of *TOC1* in *elf3-8* and *lux-6*.

Two-week-old seedlings grown under ND conditions were transferred to LL conditions at ZT0. Whole seedlings were harvested from ZT48 to ZT68 to analyze transcript accumulation. Technical duplicates were averaged. Bars indicate the standard deviation. The white and grey boxes indicate the subjective day and night, respectively. Note that these are biological replicates for Fig. 5d and 5e.

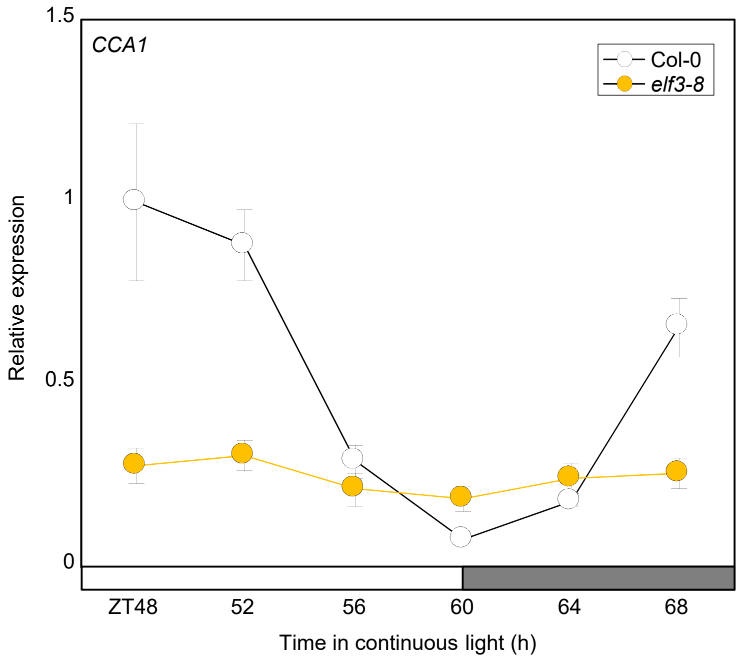

Supplementary Figure 13. Circadian expression of *CCA1* in *elf3-8*.
Two-week-old seedlings grown under ND were transferred to LL at ZT0. Whole seedlings were harvested from ZT48 to ZT68 to analyze transcript accumulation. Three independent biological replicates were averaged. Bars indicate the standard error of the mean. The white and grey boxes indicate the subjective day and night, respectively.

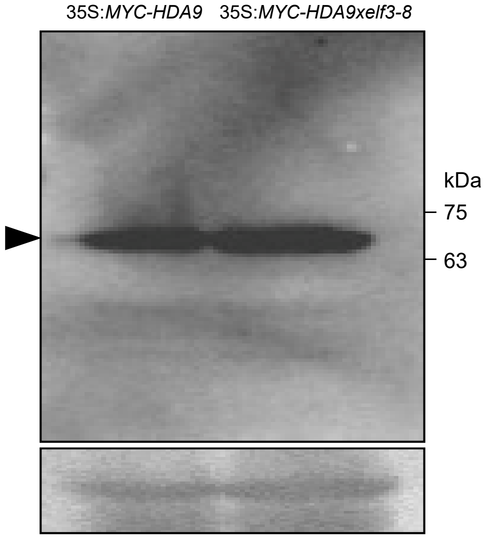

Supplementary Figure 14. Protein accumulation of HDA9 in 35S:*MYC-HDA9* and 35S:*MYC-HDA9xelf3-8*.
Ten-day-old seedlings grown under LDs were harvested for total protein isolation. The HDA9 proteins (arrowheads) were detected immunologically using an anti-MYC antibody. Part of a Coomassie blue–stained gel is shown as a loading control.

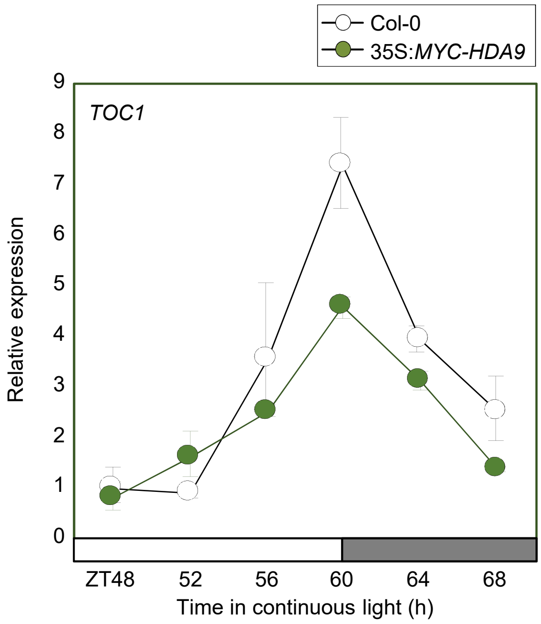

Supplementary Figure 15. Circadian expression of *TOC1* in 35S:*MYC-HDA9*.
Two-week-old seedlings grown under ND conditions were transferred to LL conditions at ZT0. Whole seedlings were harvested from ZT48 to ZT68 to analyze transcript accumulation. Technical duplicates were averaged. Bars indicate the standard deviation. The white and grey boxes indicate the subjective day and night, respectively.

Supplementary Tables

Primer	Usage	Sequence	
		F	R
eIF4a	RT-qPCR	TGACCACACAGTCTCTGCAA	ACCAGGGAGACTTGTTGGAC
CAB2	RT-qPCR	TTCCCAAGTAATCGAGCC	CCTTACCGGAGAGTTCCC
CCA1	RT-qPCR	TCTGTGTCTGACGAGGGTCGAATT	ACTTTGCGGCAATACCTCTCTGG
CCR2	RT-qPCR	CGTTATTGATTCCAAGATCA	ATCCTTCATGGCTTTCTCAT
TOC1	RT-qPCR	GTTGATGGATCGGGTTTCTC	TCATGACCCCATGCATATAG
HDA9	RT-qPCR	GCCTGCATAGCAAGATGGAA	CCGGCGTAAAGTTGACAAAA
PRR7	RT-qPCR	TGGGCCATATGGAAGCAGTA	TTTACGCACAAATTGGCCTC
PRR9	RT-qPCR	TTGGTCCTGAGCTTGGACTTT	GCTTACGCTTGATGATCCGA

Supplementary Table 1. Primers used for RT-qPCR.
The sizes of PCR products ranged from 80 to 300 nucleotides in length. F, forward primer; R, reverse primer.

Primer	Sequence	
	F	R
eIF4a	TGACCACACAGTCTCTGCAA	ACCAGGGAGACTTGTTGGAC
CCA1-(A)	GAACCTGTTTTGAGGATATAGATG	CTTTTAAGATTGAAACACTAGCGAG
CCA1-(B)	ATATAAAACTATGGCCCAAATAAGTTTAG	ATCTTGATCTAGTGGGACCTAC
ELF3-(A)	TTTAGTAAATAAGAGTGTCCAAGTG	AGAAACATAGCAAAAGCTCTAG
ELF3-(B)	AACCTCTAACATGGTAATATATCTATG	ATCATCCAATACATCACTTTTTG
LHY-(A)	TTTGGAATAATTTCGGTTATTTC	AATAGACCAGTTTTTGTTACTAACC
LHY-(B)	ATTTTGTATAGGCATTGCATG	TTGAATTAAACCCGACAAGC
LUX-(A)	ACATTGCGTGTCAGTACTGTAG	GATCTAGGTGATTGAAATTGAAATTAC
LUX-(B)	AAAAGAGATTGGTTAGGTCGGTTTG	TTTTCTTTTCCAGGAAAAAGCGC
PRR5-(A)	ATTTAAAGGTGAAAGACTGTG	AATTTTAAAGTAATTTTCCGCGG
PRR5-(B)	TTATTTGGCGTATTGGATCTC	ATTCTTTCTATAATAGTGTGG
PRR7-(A)	TTTGTCTTTTAGCACTATACGGTC	TTCTCCTTCAGTGTTCCTTC
PRR7-(B)	TTCTGGTACTAACAATGGCGGGAAAATTTAC	TAATTAACTTAATAAATGCGTTTCTAAGTCTACCC
PRR9-(A)	AGTGGGCATTTAATATTTGAAAATAATAC	AGATTTTTTTTTTTCAGCTGCTC
PRR9-(B)	CCTGCGAAGCAGAGGACCACC	AGCGGGCCTTCACTGAGCTG
TOC1-(A)	AAGAAACTATCCGAATAACTTCATGC	TTTGATGAAATTCCTCAGAGAAGATG
TOC1-(B)	AATTCAGAACCGGATAAAACCG	TTCGAGAGTAATCATTCCGGTTAG
TOC1-(C)	ATAACAGAAAAATAAAATTCTGATAATAGATTTTCTTGTTG	TCGACAGAAAGAAAAAATTGCAAATTC
TOC1-(D)	TGTTAAGGGGATAAATTAGGCGAC	GCTATGATACTTCCATGGCCAAA
TOC1-(E)	AATGGCTAAGGGTATGAAGATGCT	GAAGAGCAACAAAGAACACAGCTTA

Supplementary Table 2. Primers used for ChIP assays.
F, forward primer; R, reverse primer.
